# Comparing the Development and Viability of Horseshoe Crab Eggs Laid in Beach and Salt Marsh Habitats

**DOI:** 10.1002/ece3.72732

**Published:** 2025-12-17

**Authors:** Daniel A. Sasson, Christopher C. Chabot, Jo‐Marie E. Kasinak, Jennifer H. Mattei, Elizabeth U. Scott, Fletcher K. Hall, Michael R. Kendrick

**Affiliations:** ^1^ South Carolina Department of Natural Resources Marine Resources Institute Charleston South Carolina USA; ^2^ Department of Biological Sciences Plymouth State University Plymouth New Hampshire USA; ^3^ Department of Biology Sacred Heart University Fairfield Connecticut USA

**Keywords:** embryo, *Limulus polyphemus*, reproduction, spawning habitat

## Abstract

The horseshoe crab, *Limulus polyphemus* , has recently been found to spawn regularly in salt marshes, despite the long‐held assumption that salt marsh sediments are not conducive for embryonic development. Here, we tested the prediction that eggs laid in the salt marsh would develop more slowly and be less viable than eggs laid on beaches. We flagged the nests of spawning horseshoe crabs in the marshes and beaches of three states—South Carolina, Connecticut, and New Hampshire—and then excavated those nests one or three weeks later. We staged the excavated embryos to compare developmental progress between marshes and beaches. We found that, in general, habitat type did not affect embryonic development rate or the viability of embryos; instead, temperatures experienced by embryos drove most developmental patterns. Horseshoe crabs also buried their eggs in the marsh at shallower depths than at the beach, possibly to avoid deleterious conditions found deeper in marsh sediments. These results indicate that salt marshes are viable habitats for horseshoe crab recruitment and should be considered for the proper management and conservation of this species.

## Graphical abstract

Recent research has found that horseshoe crabs spawn regularly in salt marshes. However, it remained unclear if eggs laid in salt marsh sediments are viable. In this study, we show that eggs laid in salt marsh habitats develop similarly to those laid in beach habitats, suggesting that salt marshes are viable spawning habitats for horseshoe crabs.

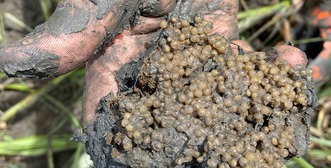

## 1 Introduction

For organisms with external embryonic development, the selection of spawning habitat is often critically important for embryonic success. Environmental conditions such as temperature, dissolved oxygen, moisture, and salinity can affect embryonic developmental rate and viability (e.g., Alderdice and Forrester 1971; Dos Santos and Nascimento 1985; Przeslawski 2004). Additionally, habitats may differ in factors such as egg predation rate (e.g., Fontaine and Martin 2006; Eggers et al. 2006) and, for intertidal organisms, the frequency of inundation (e.g., Whitmore and Dutton 1985), which may influence the survivorship of embryos. Nesting in habitats with conditions considered sub‐optimal can occur for a variety of reasons, including nesting density (Honarvar et al. 2008) and loss of traditional habitat due to anthropogenic effects (Jackson and Moser 2012). However, the actual impacts of nesting in apparently “sub‐optimal” habitats are not always quantified, making it difficult to predict the effect of this behavior at the individual and population level.

Throughout their range in the Gulf of Mexico and along the Atlantic Coast, the American horseshoe crab (HSC), *Limulus polyphemus* , plays important ecological roles in intertidal areas. When aggregated in large numbers on shore to spawn, they increase the abundance and biodiversity of coastal communities (Mattei et al. 2022). Their bioturbation of the sediment during feeding (Lee 2010) and spawning brings organisms, minerals, and nutrients to the surface (Kraeuter and Fegley 1994). They are also important links in coastal food webs, consuming small invertebrates and are in turn consumed by fish, alligators, and multiple species of sea turtles (e.g., Carmichael et al. 2004; Seney and Musick 2005, 2007). Their eggs are considered essential fuel for many migratory shorebirds, including the threatened rufa red knot ( *Calidris canutus rufa* ), which consumes mass quantities of eggs in the Delaware Bay region on their way to breeding grounds in the Arctic (e.g., Baker et al. 2004; Haramis et al. 2007).

Beaches have traditionally been considered the primary areas where *L. polyphemus* spawns. While spawning had been observed in other areas such as in peat or salt marshes, this behavior was long considered rare and maladaptive (Botton et al. 1988, 2022). However, recent studies have shown that *Limulus* spawn much more extensively in salt marshes than previously recognized (Kendrick et al. 2021; Sasson et al. 2024); in fact, both spawning adults and eggs were found in similar densities in the marsh and the beach in three regions of their range, each containing a genetically distinct population (Sasson et al. 2024), indicating that this behavior is widespread. However, it remains unclear whether spawning in marsh habitat is maladaptive; that is, is the viability lower for eggs laid in the marsh compared to those laid in the beach?

The environmental conditions in the sediment around the high tide line of sandy beaches have been thought to be most conducive to embryonic development in *Limulus* (Penn and Brockmann 1994; Jackson et al. 2008; Weber and Carter 2009). Studies have found that the combinations of temperature, oxygenation, grain sizes, and minimal hydrogen sulfides in these areas result in high viability and fast development (Botton et al. 1988; Jackson et al. 2007; Vasquez, Johnson, et al. 2015, Vasquez et al. 2017). The finding that HSCs spawn extensively in salt marshes throughout their range (Sasson et al. 2024) raises questions as to whether conditions in that habitat are suitable for embryonic development. Salt marsh sediments tend to be low in available oxygen (Bradley and Morris 1990), which can impede HSC embryonic development (Funch et al. 2016; Quigley and Santangelo 2025). High levels of moisture and hydrogen sulfides in salt marshes (e.g., Howarth 1984) may also affect development (Vasquez, Johnson, et al. 2015).

Despite these potentially deleterious conditions, HSC clutches excavated from salt marshes in South Carolina (SC) showed all stages of development, although more late‐stage embryos were found in beach habitats (Kendrick et al. 2021), suggesting slower development in marsh habitat. Embryos taken from the salt marsh also were more likely to be discolored (i.e., potentially inviable) than embryos found at the beaches, which could be due to either unfavorable conditions in the marsh or reduced fertilization of those eggs (Kendrick et al. 2021). Taken together, these findings suggest that conditions in the salt marsh may be somewhat less favorable for development than conditions on sandy beaches. However, Kendrick et al. (2021) excavated these nests through random surveys and so differences in developmental progression between habitats could be due to differences in the timing of spawning in marshes and beaches. If conditions in the marsh do result in slower embryonic development and/or reduced embryonic survivorship, these effects could have significant impacts on HSC populations, potentially limiting population growth (Sweka et al. 2007). Thus, understanding how environmental conditions and these factors differ between beach and marsh habitats may be critical for the management of the species.

In this study, we controlled for the timing of development by flagging nests in marshes and beaches during HSC spawning events and then later excavating those nests, allowing for more precise comparisons between embryonic development in the beach and marsh in SC. In addition, we did this in two additional states—Connecticut (CT) and New Hampshire (NH) where the viability of eggs in marshes had not been assessed. Since these additional states vary in the environmental conditions of their marshes and beaches and contain genetically distinct HSC populations (King et al. 2015), these investigations allowed us to also determine whether HSC egg viability in marshes is a more widespread phenomenon.

## 2 Methods

Permits to work with HSCs and excavate nests were obtained for each state, when needed. In SC, this work was conducted by South Carolina's Department of Natural Resources (SCDNR), which is authorized to conduct research, surveys, and other investigations to manage fish and marine resources in SC. In CT, scientific collection permits #SC‐20008 and #SC‐23008 to conduct research on HSCs were issued by the CT Department of Energy and Environmental Protection to Dr. Jennifer Mattei and Jo‐Marie Kasinak, respectively. In NH, permit # 2301 was issued to Dr. Christopher Chabot by NH Fish and Game to conduct this research on HSCs.

We marked nests of HSCs spawning at beaches and marshes in spring and summer of 2022 (SC) and 2023 (CT, NH) (see Table 1 for transect descriptions and sample sizes). Sites were chosen based on where HSCs were observed spawning in a previous study (Sasson et al. 2024). In SC and CT, we conducted spawning surveys at these sites during the high tides around the full and new moons, when HSCs are known to spawn in the greatest abundance. In NH, HSCs do not spawn following the lunar cycle (Cheng et al. 2016) and so spawning surveys were conducted three to four times a week.

**TABLE 1:** Locations and descriptions of transects surveyed for *Limulus polyphemus* nests in beach and marsh habitats along with sample sizes of excavated nests at each location.

State	Habitat	GPS coordinates	Substrate	Vegetation	Length of area surveyed	*N* nests excavated	
						1 week	3 weeks
South Carolina	Beach	32.428379, −80.468684	Sand	None	2.5 km	24	17
	Marsh	32.413376, −80.468351	Mud	Spartina‐dominated	80 m	26	33
Connecticut	Beach	41.173222, −73.111079	Sand and rock	None	253 m	12	15
	Marsh	41.171949, −71.107685	Mud and rock	Spartina‐dominated	408 m	10	12
New Hampshire	Beach	43.096889, −70.869694	Cobble	None	100 m	15	24
	Beach	43.130806, −70.853278	Sand/cobble	None	60 m	15	0
	Marsh	43.061056, −70.833417	Mud	Spartina‐dominated	100 m	30	0
	Marsh	43.050167, −70.83175	Mud	Spartina‐dominated	100 m	0	29

We placed HOBO loggers (MX2203, Onset Computer Corporation; Bourne, MA) at each survey site, buried to a depth of 10 cm, which corresponds to known nest depths on beaches (Penn and Brockmann 1994). These loggers continuously monitored temperatures within the sediments at the sites.

During spawning surveys, teams of two or three people walked back and forth in the spawning site looking for spawning HSCs. We considered HSCs to be actively spawning when the female buried into the sediment and remained buried for at least 1 min in SC and CT and 5 min in NH. We waited for a longer time in NH due to the difficulty for females to bury fully into the often‐rocky sediment. The nests of spawning females were marked by 3′ wire flags placed on either side of the hinge between the female's prosoma and opisthosoma. Each nest was randomly marked to indicate that its excavation would occur either one or three weeks after spawning.

We returned to the spawning sites during low tide roughly one and three weeks following spawning to excavate a subset of the nests, although the excavation of the nests did not always happen exactly one or three weeks following spawning due to logistical reasons (see Appendix Table A1 for dates of first and last nest excavation in each state). Embryos that were not collected during the excavation process were reburied where they were found. Anecdotal observations during previous fieldwork in SC suggested that HSCs may deposit their eggs at different depths in the beaches and marshes (Sasson et al. 2024), and so in SC, we took the extra step of measuring the depth at which the first eggs of a clutch appeared during excavation.

To separate the excavated embryos from the sediment, we either sieved nests in the field or brought the nests back to the laboratory for sieving. Embryos brought back to the laboratory were kept on ice during transportation and then placed in a walk‐in cooler until sieving (usually within 2 days). Keeping embryos cold greatly slows development (Lockwood 1870). Once sieved, we preserved all embryos in 95% ethanol.

We staged 50 (SC and CT) or 25 (NH) randomly selected embryos from each excavated nest. Embryos were binned into one of seven stages of development, ranging from undeveloped to advanced trilobite stage, following Kendrick et al. (2021). These stages broadly fit into the timeline of development established by Sekiguchi (1988): stage A = stages 0–14, stage B = stages 15–17, stage C = stage 18, stage D = stage 19, stage E = stages 20–1 and 20–2, stage F = stage 21a, and stage G = stage 21b. We further binned stages into early development (stages A—B), mid development (stages C—E), and late development (stages F—G). Those horseshoe crabs in late development are in the trilobite stage (i.e., they have hatched from the egg). In SC and NH, we also counted the number of discolored embryos.

We converted the temperature from the HOBO loggers into degree days for each nest. Degree days equaled the sum of the mean temperatures for the days the nest remained in the sediment prior to excavation. In NH, we lost the HOBO logger in the marsh site where nests were flagged before the data could be retrieved; however, we had a separate HOBO logger in a nearby (< 1 km) marsh site during this same time and so substituted those data to calculate marsh degree days for NH.

### 2.1 Statistical Analyses

While we surveyed multiple beach and marsh sites in SC and CT, issues with flag retention due to erosion and removal of flags by the public resulted in sufficient data from only one beach and one marsh site in each state to use in the analyses.

We compared the mean daily temperature between beach and marsh sites across states using a linear mixed model with mean daily temperature as the dependent variable, habitat, state, and their interaction as fixed effects, and date as a random effect. In NH, where we had HOBO loggers at two beach sites but only one marsh site (see above), we used temperature data from the beach site that had nests excavated after both 1 and 3 weeks and the marsh site where we were able to recover the HOBO logger. We compared degree days experienced between habitats and across states using a linear model with degree days as the independent variable and habitat, state, and their interaction as fixed effects.

We used a *t*‐test to compare the depth at which we found the shallowest eggs of marked nests across habitats.

To test how habitat type (beach and marsh) affected development, we compared the percentage of embryos from each clutch in Stages A and B for nests excavated after one week and in early, mid, and late stages of development for clutches excavated after three weeks between beach and marsh habitats. We used beta regression models in which the response variable was the percent of clutch comprised of each developmental stage (e.g., stage A, stage B, early, mid, or late development) and habitat (beach or marsh), degree day, and their interaction were fixed effects. Due to the wide temperature differences experienced by embryos across states, we ran separate models for each state. In SC and NH, we ran similar models with the percent of discolored embryos as the independent variable.

In NH, all week 3 nests in the marsh (*N* = 29) were flagged and excavated on the same dates, leading to a degree day calculation that was the same for every clutch. The beta regression models failed due to no variation in degree day for those clutches; to work around this issue, we added or subtracted 0.5 degree days to subsets of the data (*N* = 9 for each), which allowed for effective comparisons of degree days across habitats.

*T*‐tests were run using JMP 16.0. Models comparing temperatures, degree days, and development between beach and marsh habitats were run in R v4.4.3.

## 3 Results

Mean daily temperatures were higher in the beach than in the marsh in SC, but higher in the marsh than in the beach in CT and NH. Our model comparing the mean daily temperatures in beach and marshes across states did not find a significant effect of habitat (χ^2^ = 0.01, *p* = 0.90), but state (χ^2^ = 2489.0, *p* < 0.001) and the interaction of state and habitat (χ^2^ = 11.7, *p* < 0.01) were significant. Date as a random effect was also significant (χ^2^ = 288.8, *p* < 0.001).

**FIGURE 1:**
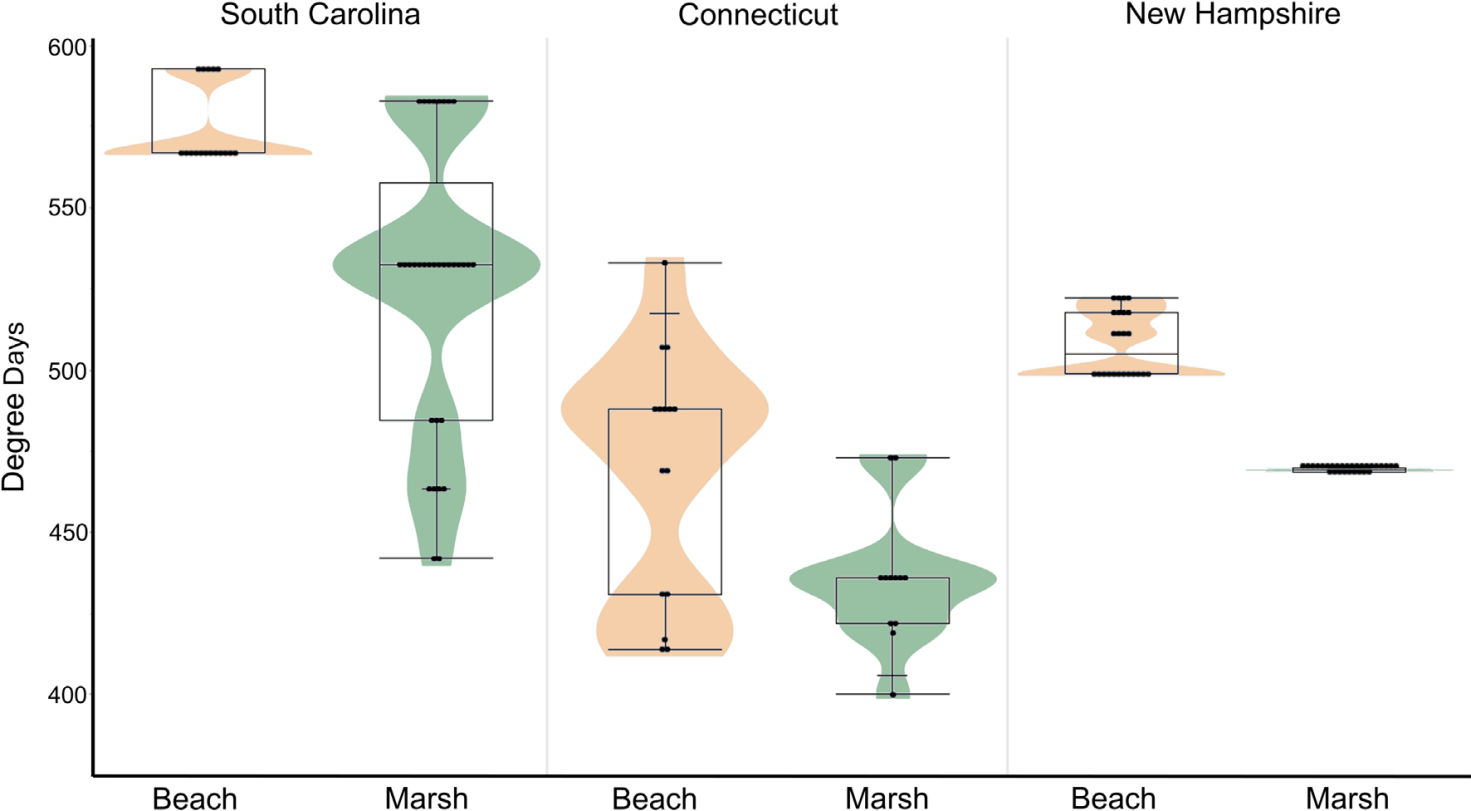
Degree days experienced by *Limulus polyphemus* embryos after three weeks of development across beach (*N*: SC = 17; CT = 15; NH = 24) and marsh (*N*: SC = 29; CT = 12; NH = 29) habitats in three states in 2022 (SC) and 2023 (CT and NH).

The degree days experienced by embryos after three weeks of development differed across habitats (*F*‐value = −70.7, *p* < 0.001) and across states (*F* = 125.1, *p* < 0.001), but the interaction between habitat and state was not significant (*F*‐value = 0.7, *p* = 0.51). In all states, the average degree days experienced by embryos was higher in the beach than in the marsh (Figure 1).

**FIGURE 2:**
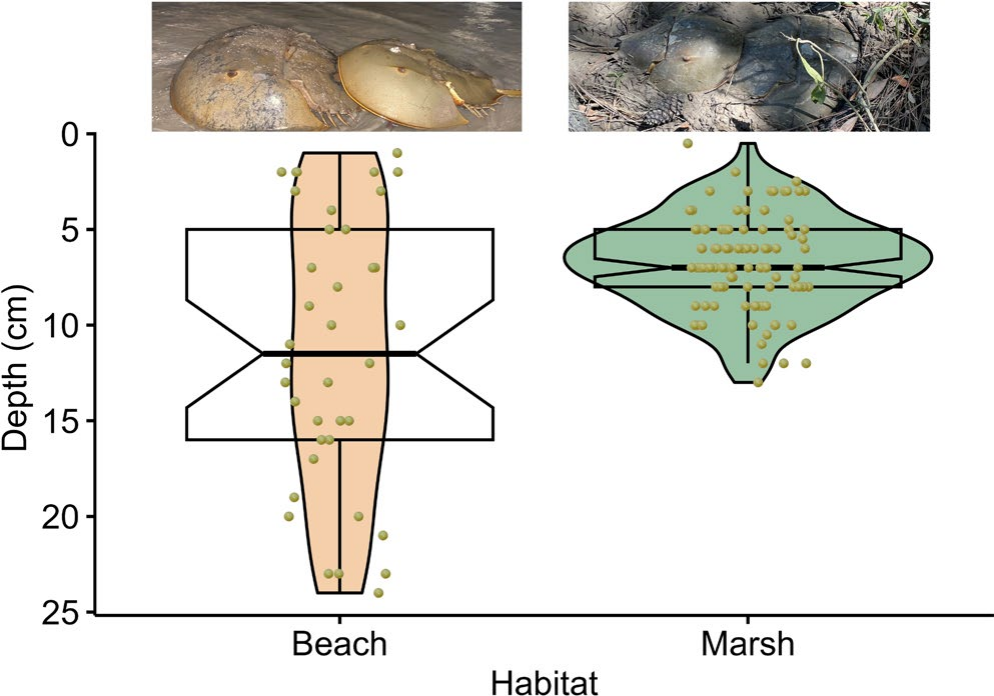
Depth of the first eggs of flagged *Limulus polyphemus* nests in South Carolina in 2022. Nests laid in the marsh (*N* = 50) were found at significantly shallower depths than nests laid on beaches (*N* = 27). The shorter but wider violin shape for nests laid in the marsh indicates less variation in the depth of marsh nests than the depth of beach nests.

In SC, nests laid in the marsh were significantly shallower than nests laid at the beach (Figure 2, *t*‐ratio = −8.0, *p* < 0.0001). On average, the first eggs were found at a depth of 6.6 cm in marsh nests and 14.3 cm in beach nests.

### 3.1 Development Across Habitats

We found no significant effect of habitat, degree day, or their interaction for the percentage of embryos in stage A or B in any state after one week of development (Table 2).

**TABLE 2:** Beta regression models of developmental progress after one week of development for *Limulus polyphemus* embryos.

	Stage A (*z*, *p*)	Stage B (*z*, *p*)
South Carolina		
Habitat	1.6, 0.12	−1.6, 0.12
Degree Days (DD)	−0.70, 0.49	0.69, 0.49
Habitat × DD	−1.5, 0.14	1.5, 0.13
Connecticut		
Habitat	0.84, 0.40	−0.81, 0.42
Degree Days	0.56, 0.58	−0.49, 0.63
Habitat × DD	−0.86, 0.39	0.84, 0.40
New Hampshire		
Habitat	0.21, 0.83	−0.23, 0.82
Degree Days	−0.57, 0.57	0.53, 0.59
Habitat × DD	−0.19, 0.85	0.21, 0.83

*Note:* No significant differences were found.

**FIGURE 3:**
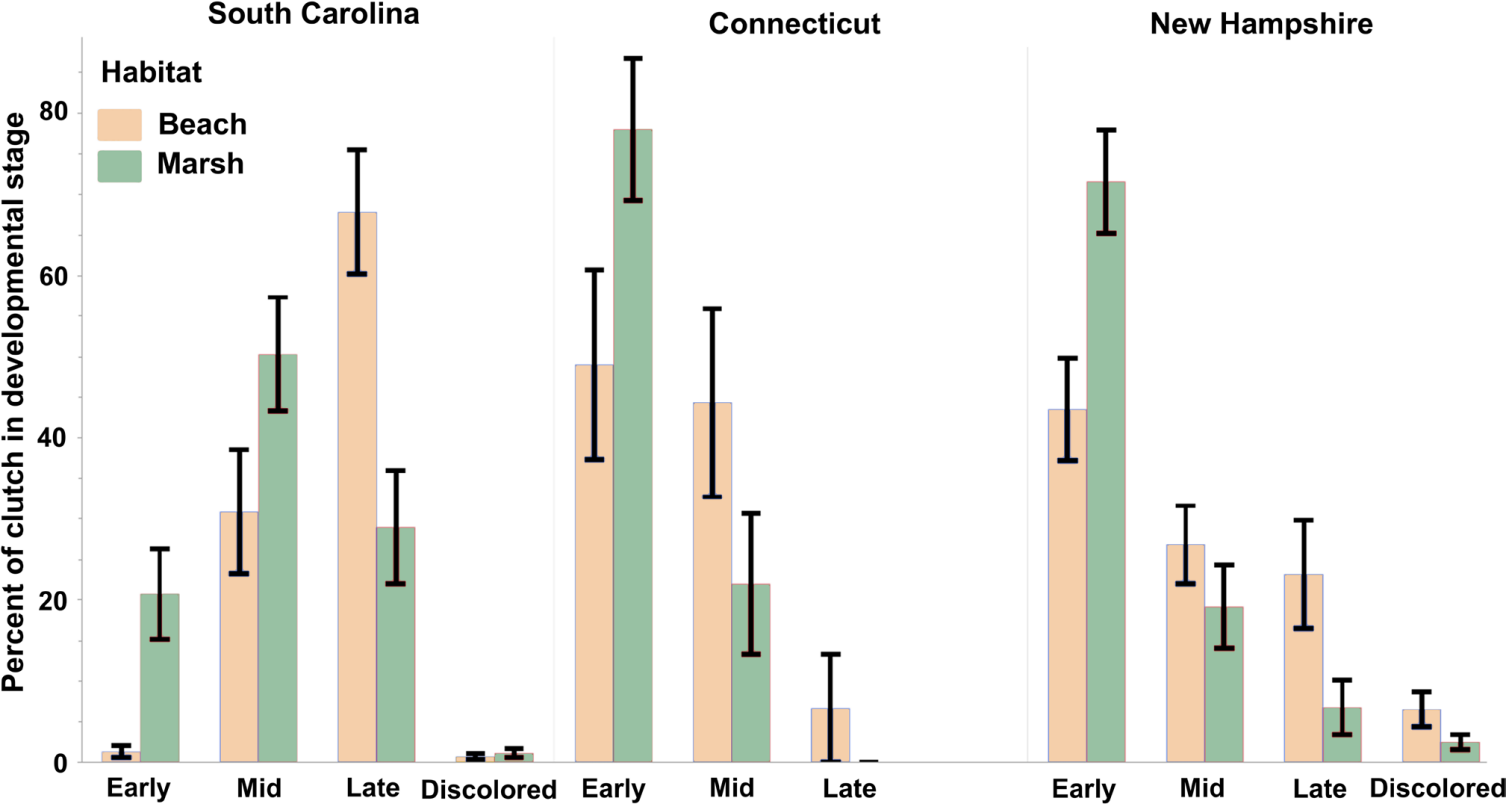
Percentage of early, mid, late‐stage, and discolored *Limulus polyphemus* embryos in clutches excavated after three weeks of development in beaches (*N*: SC = 17; CT = 15; NH = 24) and marshes (*N*: SC = 29; CT = 12; NH = 29) in three states in 2022 (SC) and 2023 (NH). Discoloration was not assessed in CT.

In SC, we found no significant effect of habitat, degree days, or their interactions for early, mid‐, and late developmental stages nor for the percentage of discolored embryos after three weeks of development (Figure 3, Table 3).

**TABLE 3:** Beta regression models of developmental progress after three weeks of development for *Limulus polyphemus* embryos.

	Early development (*z*, *p*)	Mid‐development (*z*, *p*)	Late development (*z*, *p*)	Discolored embryos (*z*, *p*)
South Carolina				
Habitat	0.78, 0.43	−1.1, 0.27	0.30, 0.77	0.56, 0.57
Degree Days (DD)	0.26, 0.79	−1.5, 0.14	1.2, 0.22	0.61, 0.54
Habitat × DD	−0.76, 0.45	1.1, 0.27	−0.32, 0.75	−0.56, 0.57
Connecticut				
Habitat	−0.63, 0.53	1.1, 0.28	−0.26, 0.80	N/A
Degree Days	**−2.0, 0.05**	**2.9, < 0.01**	−0.52, 0.61	N/A
Habitat × DD	0.68, 0.49	−1.1, 0.27	0.23, 0.82	N/A
New Hampshire				
Habitat	**2.14, 0.03**	−0.75, 0.45	−0.25, 0.81	−1.1, 0.29
Degree Days	0.24, 0.81	1.8, 0.07	−1.7, 0.10	**−2.0, 0.04**
Habitat × DD	**−2.1, 0.03**	0.75, 0.45	0.24, 0.81	1.0, 0.30

*Note:* Discolored embryos were not assessed in CT. Bold indicates statistical significance.

In CT, degree days negatively affected the percentage of embryos in early development and positively affected the percentage of embryos in mid‐development (i.e., more degree days correlated with more embryos in mid‐development) after three weeks of development (Table 3). We found no significant effect of any factor on the percentage of late‐stage embryos in CT.

In NH, we found that habitat and the interaction of habitat and degree days affected the percentage of early‐stage embryos after three weeks of development (Table 3). We found no significant effects for mid or late‐stage development, although degree days tended to positively affect mid‐stage development. We also found that degree days negatively affected the percentage of discolored embryos in the clutches.

## 4 Discussion

Salt marshes dominate the intertidal areas of the East Coast of the United States where they provide essential ecosystem services and act as critical habitat for many aquatic and terrestrial organisms (e.g., Pennings and Bertness 2001; Minello et al. 2003; Alemu et al. 2024). Recent work has shown that HSCs spawn in salt marsh habitat, an area traditionally considered inhospitable for HSC embryo development, more regularly than previously realized (Kendrick et al. 2021; Sasson et al. 2024). Despite the frequency of spawning in these habitats, it was still unclear whether embryos laid in salt marsh would develop as well as those laid on beaches. Here we show that embryos develop relatively well in the salt marshes of three regions with genetically distinct populations of HSCs (King et al. 2015). These results suggest that salt marshes are viable habitat for HSC spawning and not the potential population sinks previously feared.

In all three states, we found no indication that eggs developed more slowly in the marsh than the beach after one week. In fact, in CT and NH, very few embryos had progressed to even stage B after one week . This is somewhat surprising since our stage B roughly corresponds to stages 15–17, which Shuster and Sekiguchi (2009) indicate takes between four and five days to reach. However, that timeline was established in laboratory conditions at 30°C: the mean daily temperatures in the sediment during the first week of development were much cooler in those states (CT: beach = 18.9°C, marsh = 19.3°C; NH: beach = 14.0°C, marsh = 15.6°C). The mean daily temperature in SC was closer to those laboratory conditions (beach = 26.3°C, marsh = 23.6°C), which explains why we saw some development after one week in that state.

We saw more variation in development after three weeks, but we still found no indication that the choice of habitat itself affected the development of eggs in SC and CT. Previous work in SC suggested that eggs laid in the marsh may develop more slowly than those on the beaches (Kendrick et al. 2021), but that study did not control for the length of time the embryos experienced before excavating. With that factor accounted for in this study, we found no differences in developmental rate due to habitat. Only in NH did we find an effect of habitat after three weeks of development: our model suggested that nests laid in the beach had a significantly lower percentage of early‐stage embryos than those in the marsh. However, these findings come with an important caveat: clutches collected from the marsh were all flagged and excavated earlier in the season than those on the beach and thus had significantly lower degree days (average daily temperature: beach = 21.3 ± 2.1°C SD, marsh = 19.5 ± 1.8°C SD; average degree days: beach = 508 ± 9.9 SD, marsh = 469 ± 0 SD, see Figure 1). The significant interaction of habitat and degree days on the presence of early‐stage embryos and the tendency for degree days to positively affect the percentage of mid‐stage embryos indicate that this wide temperature difference experienced by the two treatments had a large effect on our findings.

Temperature is well known to affect embryonic development time and outcomes, such as embryonic viability and neonate size (e.g., Howe 1967; Gillooly and Dodson 2000; Gillooly et al. 2002). These effects may be especially important in ectothermic animals with external development, where environmental temperatures often vary more than in endothermic animals with internal development. We found that environmental temperatures (i.e., degree days) significantly impacted the rate of HSC embryonic development. In CT, the temperature of the sediment affected developmental rate after three weeks: clutches experiencing warmer temperatures had fewer early‐stage and more mid‐stage embryos. Despite the cooler average daily temperatures in the beach versus the marsh in CT, beach nests generally experienced higher degree days than marsh nests after three weeks of development because the majority of nests in the marsh were flagged earlier in the year than all but two beach nests. These results suggest that sediment temperatures are a main driver of differences in developmental rate between the beach and marsh, in many cases being the sole significant explanatory variable in our models. It is worth noting that the HOBO loggers were buried at a deeper depth—10 cm—than the majority of eggs excavated from the marsh, although it is unclear whether a difference in depth of a few cm would greatly have altered our results. The depth to which nests were buried was also much more varied on the beach than in the marsh, potentially suggesting greater temperature variation experienced across beach clutches than across marsh clutches. Thus, the HOBO loggers provided more of a metric to compare beach and marsh temperatures than to accurately measure the precise temperatures experienced by any one clutch. A better understanding of how temperatures vary across depths in beaches and marshes would help clarify the patterns we found here. Other environmental conditions which we did not measure such as oxygen content, moisture, and hydrogen sulfides likely also differ between the habitats (e.g., Quigley and Santangelo 2025) and previous work has investigated the role of some of these factors on embryo development in laboratory settings (Vasquez et al. 2017; Vasquez, Murillo, et al. 2015; Quigley and Santangelo 2025). Our findings suggest that any differences in development rates between habitats are due mainly to temperature rather than these other environmental conditions, but additional experiments using field‐relevant conditions would help test this hypothesis.

Marsh conditions can vary widely, and HSCs may be nesting in marsh areas more amendable to embryonic development rather than in marsh habitats with possible detrimental environmental conditions. At beach spawning sites, HSCs choose nest locations along the slope of the beach that maximize embryonic development (e.g., Penn and Brockmann 1994; Vasquez, Johnson, et al. 2015), suggesting that HSCs may be able to detect and avoid areas not conducive to development (e.g., areas with high hydrogen sulfides, Botton et al. 1988). It may be that HSCs in the marsh can similarly detect the areas at which embryos will develop best. Anecdotally, HSCs tended to nest in areas where the mud was relatively firm, which has higher oxygen levels than softer mud (Teal and Kanwisher 1961). We also found that clutches in the marsh were significantly shallower than clutches at the beach in SC: many clutches in the marsh were buried less than 5 cm below the surface. Oxygen levels in the salt marsh sediment decrease rapidly with increasing depth (e.g., Teal and Kanwisher 1961), and so the shallower depths likely provide higher rates of oxygen exchange than if the eggs were laid at typical beach depths, which range from 5 to 20 cm (Brockmann 1990). Additionally, the act of digging in the marsh itself may help to oxygenate the sediment where eggs are laid. Although it remains to be tested, these findings suggest that HSCs in the marsh, like at the beach, can detect and select areas that provide their eggs with sufficient environmental conditions for development.

Contrary to Kendrick et al. (2021), we found little evidence that eggs laid in the marsh had higher rates of discoloration (e.g., potential inviability) than eggs laid on the beach. In fact, the only significant differences in discoloration we found between the habitats was in NH where we found more discolored eggs laid at the beach than in the marsh; our models indicated that these results were significantly affected by the higher temperatures experienced by the beach clutches. Egg discoloration may be a sign that the egg is not developing—in Kendrick et al. (2021), roughly 70% of discolored eggs did not develop—which could be caused by suboptimal environmental conditions or lack of fertilization, among other factors. While we did not determine the causes of the discoloration seen in this study, our results indicate that nesting in the marsh does not increase the likelihood of embryonic inviability.

The time required for embryos to develop and emerge may have downstream impacts of viability and overall success. For instance, the longer the embryos remain in the sediment, the more time they are available for predators to find. It is unclear whether the shorebirds that consume HSC eggs regularly on the beach also feed on them in the marsh, but other nest predators, such as insects, raccoons, and even feral hogs (personal communication, Georgia DNR) are known to dig up nests and/or eat horseshoe crab eggs. Slow‐developing embryos would also emerge later, potentially leading to less time to forage on their juvenile grounds prior to winter compared to their faster‐developing cohort. However, there may also be positive effects of slow embryonic development. Slower development, which is often driven by cooler temperatures, has been shown to increase embryo viability, size at hatching, and juvenile performance in some species (e.g., Van Damme et al. 1992; Corregidor‐Castro and Jones 2021; for review, see Pechenik 1990). Warmer temperatures and faster‐developing embryos are not always ideal as this can reduce egg survival (Bouton et al. 2011) by, in part, increasing the risk of fungal or bacterial infections in aquatic invertebrates (Harvell et al. 2002; Marcogliese 2016). Furthermore, the energetic demands of oxygen uptake increase with warming temperatures (Woods 1999), which may be an additional stress on embryos laid in warmer sediments. How development time and temperature impact post‐hatching success in HSCs likely depends on numerous factors including location, quality of nearby juvenile habitat, and time in the spawning season.

As juveniles and adults, HSCs can tolerate a wide variety of environmental conditions (Shuster and Sekiguchi 2009). Here, we show that the same applies to their eggs; despite the long‐held assumption that eggs laid in muddy conditions would not develop (e.g., Botton et al. 1988), we find that eggs laid in the marsh develop similarly to those on the beach with no evidence of increased inviability. Given the large numbers of HSCs that nest in the marsh (Sasson et al. 2024) and the prevalence of the marsh habitat throughout the East Coast of the United States (e.g., Pennings and Bertness 2001), salt marshes are likely an important habitat in HSC recruitment. Quantifying the relative contribution of marsh spawning to population recruitment is essential for the proper management and conservation of this species.

## Author Contributions

**Daniel A. Sasson:** conceptualization (equal), data curation (lead), formal analysis (lead), funding acquisition (equal), investigation (equal), methodology (equal), project administration (equal), resources (equal), supervision (equal), visualization (lead), writing – original draft (lead), writing – review and editing (equal). **Christopher C. Chabot:** conceptualization (equal), data curation (equal), funding acquisition (equal), investigation (equal), methodology (equal), project administration (equal), resources (equal), supervision (equal), writing – review and editing (equal). **Jo‐Marie E. Kasinak:** data curation (equal), investigation (equal), project administration (equal), supervision (equal), writing – review and editing (supporting). **Jennifer H. Mattei:** conceptualization (equal), funding acquisition (equal), methodology (equal). **Elizabeth U. Scott:** data curation (supporting), investigation (equal), supervision (equal), writing – review and editing (supporting). **Fletcher K. Hall:** investigation (equal), writing – review and editing (supporting). **Michael R. Kendrick:** conceptualization (equal), formal analysis (supporting), funding acquisition (equal), project administration (equal), resources (equal), supervision (equal), visualization (supporting), writing – original draft (supporting), writing – review and editing (equal).

## Funding

This work was supported by U.S. Fish and Wildlife Service (Grant SC‐U2‐F21AP00690‐00).

## Conflicts of Interest

The authors declare no conflicts of interest.

## Data Availability

Data and code used in this study can be found on the Open Science Framework website (https://osf.io/ub97y/).

## Appendix A

**TABLE A1:** Dates of first flagging and last excavation of *Limulus polyphemus* nests in beach and marsh habitats with sample sizes.

State	Habitat	1 week of development			3 weeks of development		
		First nest flagged date	Last nest excavated date	*N*	First nest flagged date	Last nest excavated date	*N*
South Carolina	Beach	5/15/2022	5/23/2022	24	5/16/2022	6/6/2022	17
	Marsh	4/28/2022	5/23/2022	26	4/28/2022	6/6/2022	33
Connecticut	Beach	5/24/2022	6/21/2022	12	5/31/2023	7/7/2023	15
	Marsh	5/30/2022	6/21/2022	10	6/2/2023	7/6/2023	12
New Hampshire	Beach	5/15/2022	5/22/2022	30	6/12/2023	7/9/2023	24
	Marsh	5/16/2022	5/24/2022	30	6/7/2023	6/30/2023	29
